# Temporal Trends in Overweight and Obesity, Physical Activity and Screen Time among Czech Adolescents from 2002 to 2014: A National Health Behaviour in School-Aged Children Study

**DOI:** 10.3390/ijerph120911848

**Published:** 2015-09-18

**Authors:** Erik Sigmund, Dagmar Sigmundová, Petr Badura, Michal Kalman, Zdenek Hamrik, Jan Pavelka

**Affiliations:** 1 Institute of Active Lifestyle, Faculty of Physical Culture, Palacký University Olomouc, Tr. Miru 117, Olomouc 77111, Czech Republic; E-Mails: dagmar.sigmundova@upol.cz (D.S.); petr.badura@upol.cz (P.B.); michal.kalman@upol.cz (M.K.); jan.pavelka@upol.cz (J.P.); 2 Department of Recreation and Leisure Studies, Faculty of Physical Culture, Palacký University Olomouc, Tr. Miru 117, Olomouc 77111, Czech Republic; E-Mail: zdenek.hamrik@upol.cz

**Keywords:** excess body weight, obesity, physical activity, television (TV) watching, computer (PC) use, school-aged children, HBSC study

## Abstract

This study examines trends in overweight and obesity, physical activity (PA) and screen time (ST) among Czech adolescents over a recent 12-year study period. Nationally representative samples consisted of 19,940 adolescents (9760 boys and 10,180 girls) aged 10.5–16.5 years from the Czech Health Behaviour in School-aged Children (HBSC) questionnaire-based surveys conducted in 2002, 2006, 2010 and 2014. Trends in the prevalence of overweight/obesity, meeting the recommendations for moderate-to-vigorous PA (MVPA) (≥60 min per day of MVPA) and excessive ST (>2 h per day) were estimated using logistic regression. Significant increases (*p* < 0.001) in the prevalence of overweight/obesity between the years 2002 and 2014 were evident for both adolescent boys (18.3%_2002_–24.8%_2014_) and girls (8.3%_2002_–11.9%_2014_). Compared to 2002, in 2014 significant decreases (*p* < 0.001) in meeting MVPA recommendations were observed among boys (32.2%_2002_–25.6%_2014_) and girls (23.2%_2002_–19.2%_2014_). Moreover, in boys we observed significant increases (*p* < 0.001) in excessive ST on weekdays (75.1%_2002_–88.8%_2014_), as well as on weekends (78.3%_2002_–91.9%_2014_) between the years 2002 and 2014. Increases in overweight/obesity with concomitant decreases in PA provide evidence in support of the current and upcoming efforts of government and commercial organizations in implementing interventions aimed at reducing excessive body weight among Czech adolescents.

## 1. Introduction

The prevalence of childhood obesity has risen substantially over recent long-term (20–30 years) [1,2], medium-term (8–12 years) [3,4,5] and short-term (2 years) time periods [6] in developed as well as developing countries [1,2,3,4,5,6,7,8]. In addition, the fact that in developing countries the peak prevalence of obesity has moved from older adults to younger age cohorts is of concern [1]. Although other trend studies [9,10,11,12,13,14] point to a stable prevalence of childhood overweight and obesity in developed countries over the past 15 years, the apparent plateau of childhood overweight and obesity in those countries is still at an unacceptably high level and constitutes a serious public health issue.

The latest studies on the prevalence of overweight and obesity in children and adolescents, including data from the Czech Republic (19.1%–26.3% in boys and 9.5%–21.0% in girls) [1,5,14], document an above-average prevalence of overweight and obesity in Central European countries and Denmark, Japan, France, Norway and Sweden, but a below-average prevalence in Australia, Canada, Ireland, New Zealand, Spain, the United Kingdom (UK) and the United States of America (US) [1,4,5,8,14]. Analogously, as in Australia, Canada, Denmark, France, the Netherlands, New Zealand, Norway, Spain and Sweden, but unlike the UK and US, in the Czech Republic a higher proportion of overweight/obesity was revealed in boys than in girls [1,4,5,8,14]. Moreover, the difference in the prevalence of overweight/obesity between boys and girls in the Czech Republic [1,5,14] is higher than in Australia, Canada, Denmark, Japan, France, the Netherlands, New Zealand, Norway, Sweden, and the US.

Childhood obesity has arisen due to a complex array of interactions among multiple behavioural, biological and environmental factors that adversely affect the long-term energy balance [15]. Lifestyle factors such as a low level of physical activity (PA) and excessive sedentary behaviour have been directly associated with childhood overweight and obesity [16,17,18,19]. To provide evidence in support of preventive efforts, it is desirable to gain more insight into the trends in overweight/obesity, PA (moderate-to-vigorous (MVPA) and vigorous (VPA)) and sedentary behaviour (screen time) as well as changes in how these behaviours and health outcomes correlate with one another.

Variations in temporal trends in adolescent PA have been documented cross-nationally for the years 2000 to 2012. While in the cases of Portuguese [7], Czech [20,21], Danish, Swiss, Italian [21] and Mozambican [22] adolescents a decrease in self-reported [7,21,22] and pedometer-determined PA [20] was observed, a stabilized pedometer-determined PA level was recorded in Swedish adolescents [23]. In Iceland [24], Belgium, Finland, France and Germany [21] increases in adolescents’ self-reported PA were reported. Lower levels of PA were reported by Czech adolescents; Swedish adolescents achieved a higher daily step count than Czech adolescents of the same age both in 2000 and in 2008 [20,23]. Furthermore, while in 2002 Czech and American 11- to 15-year-old adolescents achieved similar levels of moderate-to-vigorous PA (MVPA), in 2010 US adolescents reported higher levels than Czech adolescents in the number of days per week with at least 60 min of MVPA [13,20].

With regard to screen time (ST), the amount of time devoted to electronic entertainment and computer (PC) use rose sharply during the first decade of the twenty-first century across developed and developing countries around the world [5,7,20,25,26,27]. Over the past decade, rates of daily ST (including TV/DVD watching, playing games on a computer or games console, using a PC) have been quite stable in many countries such as Brazil [27], the Czech Republic [5,20], China [28], Germany [26], Portugal [7] and the US [13]. In addition, changes in the types of ST reported have been recorded in the same period. TV/DVD watching was replaced by games played on a computer or games console and time spent using computers among boys and girls throughout the entire adolescent period [5,7,13,20,25,26,27]. The repeatedly observed increase in ST at weekends compared with weekdays among Brazilian [27], Czech [5] and German [26] adolescents is considered to be an especially disturbing finding.

From a public health perspective, it is important to study changes in trends of overweight and obesity among adolescents, because the financial costs associated with the treatment of the related diseases in adulthood are high and continually rising [29,30]. Focused study of temporal changes in adolescents’ overweight and obesity and their correlates (including MVPA and screen time) is also important since overweight adolescents have an 83% chance of becoming overweight adults, as evidenced by longitudinal study findings [31]. Moreover, long-term prospective follow-up of Swedish adults indicate that cardio-metabolic risk in adulthood (average 43 years) may be caused by sustained TV watching over the life course and TV watching during early life [32]. TV watching in adolescence may also represent a sensitive period for the manifestation of metabolic syndrome in mid-adulthood [32]. Conversely, those who maintained their self-reported MVPA at a frequency of at least twice a week from adolescence (age 13–19 years) to young adulthood (age 23–31 years) demonstrated a significantly lower cardiovascular disease risk and better mental health than individuals failing to be physically active in MVPA at least twice a week [33].

Discovery of trends and mutual associations between overweight/obesity, MVPA, VPA and ST can provide insight into the lifestyles of adolescents and can help formulate public health-related recommendations to reduce excess body weight. Although there are local school-based PA interventions shown to effectively reduce overweight and obesity in 8- to 12-year-old Czech children [34,35], at the national level efforts to reduce childhood obesity have been less successful. Furthermore, across Central and Eastern Europe, rapid increases in the rates of coronary heart disease and stroke have been projected, predicting 110,000 cumulative incidence cases in 2050 compared to a 100,000 population in Croatia, the Czech Republic and Romania in 2010 [36]. Previous studies [5,21] on trends of overweight and obesity, PA and ST in Czech adolescents between 2002 and 2010 have still left unanswered questions. These surround whether the gradual increase in the prevalence of overweight and obesity (only in boys), as well as the increase in the daily amount of ST (boys and girls), and will continue in the directions observed in the recent past.

The main objective of this analysis of the Czech national HBSC study is to describe time trends in adolescents’ overweight/obesity, physical activity (MVPA and VPA) and screen time (TV and PC) over four recent cycles of this study (2002, 2006, 2010 and 2014). Specific objectives were to:

(i) Determine whether a gradual increase in the prevalence of overweight/obesity in boys continued in 2014, and also assess the direction of trends in the prevalence of overweight/obesity in girls;
(ii) Describe temporal trends in the achievement of PA recommendations (MVPA—at least 60 min per day, VPA – at least 30 min per week and four or more days of the week);
(iii) Consider whether a rapid increase in ST in boys and girls on weekdays and at weekends continued in the 2014 cycle;
(iv) Identify correlates of overweight/obesity that potentially contributed to the high prevalence of overweight and obesity among Czech adolescents in the 2002–2014 period.

## 2. Methods

### 2.1. Study Design

This study is based on four cycles of the Czech Health Behaviour in School-aged Children (HBSC) study. The Czech Republic is one of the 43 countries and regions from across Europe and North America that, in four-year intervals, participated in a cross-national survey of 11-, 13- and 15-year-old adolescents based on an internationally agreed protocol [37]. The HBSC study focuses on the description of health, well-being and health behaviour and their social determinants [37]. The study is based on a self-administered questionnaire that is completed in public schools [38]. A detailed description of the theoretical framework, aims and methodological development of the HBSC study can be found online at www.hbsc.org or elsewhere [37,38].

### 2.2. Sample

The sample consisted of reports from 19,940 adolescents (9760 boys and 10,180 girls) from the 5th, 7th and 9th grades of primary schools in the Czech Republic. Across the four cycles, sample sizes were as follows: in 2002 (n = 5012), 2006 (n = 4774), 2010 (n = 4404) [5] and 2014 (n = 5750) (Table 1). The 5th (7th and 9th, respectively) grade involves adolescents in the age range 10.5–12.49 years (12.50–14.49 years and 14.50–16.49 years, respectively. In each year of data collection, a single-stage cluster sampling approach was used to obtain the participants in accordance with the international protocol [37]. Schools were the primary sampling unit, and they were stratified by the region and administrative district. The Czech HBSC excludes students in special needs schools, private schools, youth in detention centers, and youth and students who are home schooled. The school response rate varied among surveys from 75% to 89% and the pupil response rate in all four waves of the survey exceeded 80%. The basic sample characteristics are presented in Table 1 separately for each survey year and gender. The study was approved by the Ethical Committee of the Faculty of Physical Culture, Palacký University Olomouc (No. 17/2013).

### 2.3. Instrument and Variables

Data collection was based on the completion of a standardized self-report questionnaire during a single class, with a researcher administering and supervising. During one morning school hour (45 min), the researcher administered the questionnaires in the classroom and instructed students on how to fill them in. To ensure anonymity, after completing the survey students were instructed to seal the questionnaire in an envelope and hand it over to the researcher. Efforts were made to retain a core of identical items in each wave of the survey to allow trends to be observed [38].

#### 2.3.1. Weight Status

Self-reporting of own body height and weight in adolescents is considered valid for Body Mass Index (BMI) estimates of overweight and obesity in epidemiological studies [39]. The actual body height and weight of students were self-reported in the HBSC questionnaire with an accuracy of 0.5 cm and 0.1 kg. The BMI (kg/m^2^) was calculated as body weight (kg) divided by body height (m) squared. Obese, overweight and normal body mass in children were classified using the World Health Organization (WHO) percentile BMI charts for girls and boys between the ages of 5 and 19 [40]. Overweight and obesity were represented by 85%–97% and >97%, respectively, on age-differentiated BMI charts [40]. The chronological age of adolescents, which was used to compute the age-differentiated BMI, was calculated as the difference between the date of application of the HBSC questionnaire and the self-reported month and year of birth of the student.

#### 2.3.2. Physical Activity

For the purposes of trends analyses, we studied two levels of self-reported MVPA (moderate-to-vigorous physical activity) and VPA (vigorous physical activity). MVPA was determined by the following question: “Over the past 7 days, on how many days were you physically active for a total of at least 60 min per day?” with eight response categories ranging from “0 days” to “7 days”. For the purposes of clinical practice with adolescents the question was originally developed more than ten years ago [41] and validated against seven-day continuous measurement with a accelerometer (r = 0.40, *p* < 0.001) with a substantial test-retest stability (Intraclass correlation coefficient (ICC = 0.77)) [41]. Current studies [42,43] support the usability of this question for the classification of MVPA in the past 7 days in adolescents (r = 0.49, *p* < 0.01 correlation with seven-day continuous measurement with an Actigraph accelerometer) [42] with almost perfect test-retest stability in Chinese (ICC = 0.82) [43] and Polish (ICC = 0.98) [44] 11–15-year-old adolescents. For the analysis of trends in meeting the current MVPA recommendations (≥60 min per day) [45,46] a dichotomous outcome variable was created. Adolescents who indicated that they were active for at least 60 min on each of the past seven days were classified as meeting MVPA recommendations.Main text paragraph.

The VPA item has been included in the HBSC survey since 2006. VPA was estimated using two questions on the frequency and volume of VPA outside school hours: (1) “How often do you usually exercise in your free time so much that you get out of breath or sweat?” with seven response categories: “Every day”, “4 to 6 times a week”, “2 to 3 times a week”, “Once a week”, “Once a month”, “Less than once a month” and “Never”. (2) “How many hours a week do you usually exercise in your free time so much that you get out of breath or sweat?” with six response categories: “None”, “About half an hour”, “About 1 h”, “About 2 to 3 h”, “About 4 to 6 h” and “About 7 h or more”. Assessment of self-reported VPA during the past seven days has also been validated against measurements using the Computer Science and Applications (CSA) accelerometer (r = 0.36, *p* < 0.01) [41]. The test-retest stability of both VPA questions showed moderate agreement (ICC_1_ = 0.68, ICC_2_ = 0.57) [43]. In line with existing precedents [46,47], we dichotomized outcome variables and combined both the questions into a single dichotomous outcome variable for VPA as follows: at least 30 min per week of VPA and four or more days of the week, *versus* less than four days of the week of any combination of duration of VPA.

#### 2.3.3. Screen time (ST)

In all four waves of the HBSC survey, attention was paid to two ST components—television watching (TV) and computer use (PC). TV was self-reported using one question divided separately into weekdays and weekends: “About how many hours a day do you usually watch television (including DVDs and videos) in your free time?” with nine response categories: “None at all”, “About half an hour a day”, “About 1 h a day”, “About 2 h a day”, “About 3 h a day”, “About 4 h a day”, “About 5 h a day”, “About 6 h a day” and “About 7 or more hours a day”. During the years 2006–2014 the time spent using a PC was determined by two questions divided separately into weekdays and weekends: (1) “About how many hours a day do you usually play games on a computer or games console (PlayStation, Xbox, GameCube *etc.*) in your free time?” and (2) “About how many hours a day do you usually use a computer for chatting online, Internet, emailing, homework *etc.* in your free time?”. Both of these PC questions used the same categories of possible answers as TV watching. In 2002, the PC time was investigated using a single item, which aimed to investigate both the issues—playing games on a computer or console and using a computer for chatting online, Internet, emailing, homework *etc.* in free time. Self-reported questions on ST for the past seven days have been validated against a seven-day log among 11- to 15-year-old adolescents with a suitable validity on weekdays (r = 0.39–0.46, *p* < 0.001) and at weekends (r = 0.37–0.47, *p* < 0.001) [48,49]. The test-retest stability of self-reported ST questions has been repeatedly verified for both weekdays (ICC_TV_ = 0.54–0.72, ICC_PC_ = 0.33–0.82) and weekend days (ICC_TV_ = 0.58–0.68, ICC_PC_ = 0.33–0.66) [43,44,48,50]. Validation studies [48,49] indicate that adolescents do not have a systematic tendency to overestimate or underestimate the amount of daily ST, and current TV and PC questions appear to have adequate reliability [43,44,48,50] and validity [48,49] for the surveillance of adolescents. In line with the recommendations of previous studies on ST [51,52,53], we dichotomized the outcome variables for TV and PC as follows: two or fewer hours *versus* more than two hours of ST per day. Spending two or more hours a day watching TV or using a PC is classified as excessive [5,13,51,52,53].

### 2.4. Data Treatment and Statistical Analyses

Because previous trend studies [5,20] have demonstrated clear differences in excess body weight, MVPA and sedentary behaviour between adolescent boys and girls, the prevalence of overweight/obesity, as well as the prevalence of respondents meeting the MVPA, VPA and ST recommendations, are presented separately for boys and girls throughout all the survey years. Descriptive characteristics include percentages in each output variable category, means (hours per day of TV and PC) and confidence intervals (CI). Time trends of output variables (except VPA) between the years 2002 and 2014 were estimated using the logistic regression Enter method with the 2002 survey as the reference category. Trends in VPA were analysed between 2006 and 2014 using the logistic regression Enter method with the reference category of 2006. The logistic regression Enter method was also applied to uncover correlates of overweight/obesity of Czech adolescents. Regression parameters are based on odds ratio (OR) with 95% CI. All statistical analyses were performed using the SPSS v21.0 software (IBM SPSS, Inc., Chicago, IL, USA). The data were analysed in total for all primary schools because the TwoStep cluster analysis found no indicator for clustering by school.

## 3. Results

The present study included data from 19,940 adolescents, of which 48.95% were boys. Participants’ ages ranged from 10.5 to 16.5 years (32.21% were from the 5th grade, 33.57% were from the 7th grade, and 34.22% were from the 9th grade) (Table 1).

**Table 1:** Descriptive characteristics of the samples, Health Behaviour in School-aged Children study, Czech Republic 2002–2014.

	2002		2006		2010		2014	
	Boys	Girls	Boys	Girls	Boys	Girls	Boys	Girls
	(n = 2412)	(n = 2600)	(n = 2410)	(n = 2364)	(n = 2135)	(n = 2269)	(n = 2803)	(n = 2947)
	%	%	%	%	%	%	%	%
**School grade** ^§^								
5th grade	34.3	33.3	31.7	31.5	33.7	31.2	30.9	31.4
7th grade	32.3	33.8	33.4	33.7	31.3	34.7	34.3	34.5
9th grade	33.4	32.8	34.9	34.8	35.0	34.2	34.8	34.1
**Weight status** ^#^								
Normal weight	81.7	91.7	77.6	83.7	73.7	88.0	75.3	88.1
Overweight/obese	18.3	8.3	22.4	16.3	26.3	12.0	24.7	11.9
**Daily MVPA** *								
≥60 min	32.2	23.2	27.4	17.9	28.3	19.2	25.6	19.2
**VPA per week** ^†^								
≥4 times 30 or more minutes	-	-	45.7	24.4	44.9	30.3	42.0	33.6
**Daily screen time** ^‡^								
>2 h per weekday	75.1	61.3	86.0	73.4	87.6	81.4	88.7	76.6
>2 h per weekend day	78.3	65.1	88.6	79.8	89.4	84.8	91.8	83.7

^§^ 5th (7th, and 9th) grade includes adolescents in the age range 10.5–12.49 years (12.50–14.49 years and 14.50–16.49 years); ^#^ Overweight and obesity is classified according to the WHO growth reference for school-aged children, where overweight and obesity were represented by 85%–97% and >97%, respectively, on age-differentiated Body Mass Index charts [38]; * MVPA—moderate-to-vigorous physical activity (≥60 min per each of the past seven days [42,43]); ^†^ VPA—vigorous physical activity (≥30 min of VPA and four or more days of the week [43,45]); ^‡^ Screen time—amount of time spent watching television or using a computer (screen time >2 h per day on each of the past seven days is classified as excessive [49,50,51]).

### 3.1. Trends in Excess Body Weight (Overweight and Obesity)

Trends in the prevalence of excess body weight between the years 2002 and 2014 were consistent for girls and boys (Table 2). There were some differences evident by gender in the different study cycles. The group of girls, regardless of school grade category, reached their peak of prevalence of overweight and obesity in 2006, which was followed by a levelling off in this prevalence (9th grade) or even a decline (5th and 7th grades) in the next two survey cycles. In the group of boys from the 5th, 7th and 9th grades a gradual increase in the prevalence of overweight and obesity was observed until 2010, which was followed by a slight decline in the prevalence of excess body weight.

However, when comparing 2014 to 2002 we found a significant increase in the prevalence of overweight and obesity in both adolescent boys and girls in all age categories (Table 2). The highest proportion of children with excessive body weight within groups of girls and boys has been repeatedly observed in the youngest age category.

### 3.2. Trends in Attainment of the Physical Activity Recommendation

For the two PA outcome variables (MVPA and VPA), we found different trends between 2002 and 2014, and 2006 and 2014, respectively (Table 2). Between 2002 and 2014, we found an oscillating decline in the proportions of adolescents of both genders who met the recommendations of at least 60 min of MVPA a day. We observed a continuous decrease in the ratio of boys meeting the VPA recommendations between 2006 and 2014. Reversely, the ration of girls who met the VPA recommendations increased between 2006 and 2014. Only among 7th grade girls was the declining trend in meeting the MVPA recommendations between 2002 and 2014 not statistically significant (Table 2). Within the group of girls, the 5th graders were repeatedly assessed as having the highest proportion of girls meeting the MVPA recommendations between 2002 and 2014. Of all the boys, the most significant decline in meeting the VPA recommendations between 2006 and 2014 was observed among 5th grade boys. Among all the girls, in those attending the 5th grade we recorded the smallest increase in the percentage of individuals meeting the VPA recommendations between 2006 and 2014 (Table 2).

### 3.3. Trends in Non-Excessive Screen Time

When comparing non-excessive daily amount of ST between 2002 and 2014, in all age groups of both genders we observed a significant reduction in the proportion of adolescents reaching a maximum of 2 h of ST per day on weekdays, as well as on weekends (Table 2). Different trend trajectories in the 2002–2014 periods in the proportion of a non-excessive daily amount of ST were observed between boys and girls.

**Figure 1:**
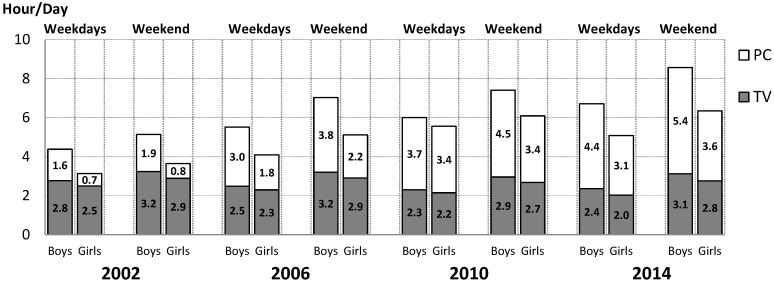
Mean daily hours of screen time among Czech adolescents between 2002 and 2014 (TV: television watching, PC: computer use).

For the whole group of boys we found a steady decrease in the proportion of adolescents with a non-excessive daily amount of ST between 2002 and 2014 on weekdays as well as at weekends, while for girls we observed an obvious convex course in the proportion of adolescents with a non-excessive daily amount of ST between 2002 and 2014, with the lowest point in 2010 (Table 2). We also observed a slight decrease in TV watching and a notable increase in PC use, both on weekdays and weekend days between 2002 and 2014 (Figure 1). While the overall decrease in the TV watching of adolescents between 2002 and 2014 ranged from 0.4 to 0.5 h per weekday (or 0.1 h per weekend day), the considerable increase in PC use ranged from 2.4 to 2.8 h per weekday (or from 2.8 to 3.8 h per weekend day) (Figure 1).

**Table 2:** Trends in prevalence of overweight/obesity, PA and ST recommendations in Czech adolescents between 2002–2014.

	2002	2006	2010	2014	2014 *vs.* 2002		
Odds ratio to reach the variables ^1−5^						95% CI	
	% ^a^	% ^a^	% ^a^	% ^a^	OR	Lower	Upper
**Overweight/obese** ^1^							
Boys	18.3	22.4	26.3	24.7	1.46 ***	1.28	1.68
5th grade	21.5	30.1	30.6	27.8	1.41 **	1.12	1.77
7th grade	17.3	21.2	27.0	26.6	1.73 ***	1.37	2.20
9th grade	16.0	16.6	21.8	20.6	1.36 *	1.06	1.74
Girls	8.3	16.3	12.0	11.9	1.49 ***	1.24	1.78
5th grade	10.1	22.7	15.3	14.0	1.45 *	1.08	1.94
7th grade	8.6	15.5	10.6	11.3	1.36 *	1.00	1.86
9th grade	6.2	11.5	10.7	10.7	1.80 ***	1.27	2.55
**MVPA recommendations** ^2^							
Boys	32.2	27.4	28.3	25.6	0.72 ***	0.64	0.82
5th grade	36.2	25.7	29.0	29.5	0.74 **	0.60	0.91
7th grade	32.6	28.6	30.6	27.2	0.77 *	0.63	0.95
9th grade	27.9	27.9	25.5	20.4	0.66 ***	0.53	0.83
Girls	23.2	17.9	19.2	19.2	0.79 ***	0.69	0.90
5th grade	28.5	19.7	24.1	24.2	0.80 *	0.65	0.99
7th grade	22.5	17.6	19.4	20.1	0.87	0.69	1.08
9th grade	18.4	16.5	14.9	13.6	0.70 **	0.54	0.90
**Non-excessive ST weekday** ^3^							
Boys	24.9	14.0	12.4	11.2	0.38 ***	0.33	0.45
5th grade	32.2	19.0	19.6	15.4	0.38 ***	0.30	0.48
7th grade	21.1	11.3	9.4	11.2	0.47 ***	0.36	0.62
9th grade	21.1	11.9	8.2	7.6	0.31 ***	0.23	0.41
Girls	38.7	26.5	18.5	23.4	0.48 ***	0.43	0.54
5th grade	44.2	33.4	28.4	34.3	0.66 ***	0.55	0.80
7th grade	34.1	23.5	13.5	23.2	0.59 ***	0.48	0.72
9th grade	37.9	23.3	14.6	13.7	0.26 ***	0.21	0.33
**Non-excessive ST weekend day** ^4^							
Boys	21.7	11.4	10.6	8.1	0.31 ***	0.27	0.38
5th grade	28.1	13.7	15.8	12.0	0.35 ***	0.27	0.45
7th grade	20.1	10.6	9.0	7.1	0.30 ***	0.22	0.41
9th grade	16.7	10.0	7.0	5.7	0.30 ***	0.22	0.42
Girls	34.9	20.1	15.3	16.3	0.36 ***	0.32	0.41
5th grade	39.6	27.1	25.6	26.4	0.55 ***	0.45	0.67
7th grade	31.1	17.0	11.2	13.4	0.34 ***	0.27	0.43
9th grade	34.0	16.9	10.0	10.1	0.22 ***	0.17	0.28
**VPA recommendations** ^5^					**2014** *vs.* **2006**		
Boys	-	45.8	44.9	42.2	0.86 **	0.77	0.96
5th grade	-	45.4	46.3	39.4	0.78 *	0.64	0.96
7th grade	-	47.1	46.1	42.6	0.83	0.69	1.01
9th grade	-	44.8	42.6	44.4	0.98	0.81	1.18
Girls	-	24.4	30.3	33.6	1.57 ***	1.39	1.77
5th grade	-	30.2	37.1	34.4	1.22	0.99	1.50
7th grade	-	23.0	30.7	36.2	1.90 ***	1.54	2.35
9th grade	-	20.5	23.6	30.2	1.67 ***	1.35	2.08

MVPA: moderate-to-vigorous physical activity; ST: screen time; VPA: vigorous physical activity; OR: odds ratio; CI: 95% confidence interval; logistic regression Enter method (LR): ^1^ OR of being overweight/obese, ^2^ OR of achieving MVPA recommendations (≥60 min of MVPA per day), ^3,4^ OR of meeting ST recommendations (≤2 h per weekday or ≤2 h per weekend day), ^5^ OR of achieving VPA recommendations (≥30 min of VPA and 4 or more days of the week); % ^a^: percentage of adolescents: who are overweight/obese (LR ^1^), who reach MVPA recommendations (LR ^2^), who meet ST recommendations (LR ^3^ or LR ^4^); who reach VPA recommendations (LR ^5^); LR ^1−4^: reference group is a cohort of 2002, LR ^5^: reference group is a cohort of 2006; * *p* < 0.05, ** *p* < 0.01, *** *p* < 0.001.

### 3.4. Correlates of Excessive Body Weight (Overweight/Obesity)

Logistic regression analyses revealed that being a girl *vs.* boy, being in the oldest relative to youngest age category, and reporting MVPA recommendations of at least 60 min per day were all highly protective for overweight and obesity (*p* < 0.001) (Table 3).

**Table 3:** Correlates of overweight/obesity in randomized sample of Czech adolescents.

	Overweight/Obese			
			95% CI	
	%	OR	Lower	Upper
**Year of data collection**				
2014	18.2	Ref.		
2010	19.0	1.06	0.95	1.18
2006	19.4	1.09	0.98	1.21
2002	13.1	0.69 **	0.62	0.77
**Gender**				
Boys	22.9	Ref.		
Girls	12.0	0.46 **	0.42	0.50
**School grade ^1^**				
5th grade	21.2	Ref.		
7th grade	17.0	0.72 **	0.66	0.79
9th grade	14.2	0.58 **	0.53	0.64
**MVPA recommendations ^2^**				
<60 min of MVPA per day	17.6	Ref.		
≥60 min of MVPA per day	15.9	0.81 **	0.74	0.89
**ST recommendations weekday ^3^**				
>2 h per week day	18.3	Ref.		
≤2 h per week day	14.0	0.86 *	0.77	0.97
**ST recommendations weekend day ^3^**				
>2 h per weekend day	18.1	Ref.		
≤2 h per weekend day	14.1	0.90	0.80	1.02

MVPA: moderate-to-vigorous physical activity; ST: screen time; OR: odds ratio (logistic regression Enter method); CI: 95% confidence interval; %: percentage of overweight/obese adolescents (overweight and obesity represented 85%–97% and >97%, respectively, on age-differentiated Body Mass Index charts [38]); ^1^ School grades of primary schools include adolescents in the age ranges as follows (5th: range from 10.5 to 12.49 years, 7th: range from 12.50 to 14.49 years, and 9th: range from 14.50 to 16.49 years; ^2^ MVPA recommendations (achieve ≥60 min per each of the past seven days [42,43])); ^3^ ST recommendations (≤2 h per weekday or weekend day is classified as non-excessive [49,50,51]); * *p* < 0.01, ** *p* < 0.001.

Spending less than two hours per weekday on ST was also significantly (*p* < 0.01) associated with reduced odds of being overweight or obese. Moreover, the odds of adolescents being overweight/obese in 2014 were significantly higher than when 2002 reported levels (*p* < 0.001) yet similar to 2006 and 2010 data collection waves (Table 3).

## 4. Discussion

To the best of our knowledge, this is the first study from the countries of Central and Eastern Europe to analyse temporal trends in overweight and obesity and their correlates (physical activity and screen time) among adolescents in the 12-year period of 2002–2014. The Czech Republic has undergone a substantial economic transformation during this time. Similar to other Central and Eastern European countries undergoing such transformations, there is a tendency for such countries to mimic trends in obesity and its behavioural determinants observed in more Western countries, *i.e.*, decreased levels of PA and increased prevalence of overweight and obesity [54,55]. Therefore, from a public health perspective it is important to monitor trends in overweight/obesity and their correlates in such countries.

We investigated whether the continual increase in the prevalence of overweight/obesity among boys also persisted in 2014 and assessed the direction of trends in the prevalence of overweight/obesity in girls. The overall significant increase in the prevalence of overweight and obesity in Czech adolescent boys (18.3%_2002_ to 24.8%_2014_, +6.5%, *p* ≤ 0.001) and girls (8.3%_2002_ to 11.9%_2014_, +3.6%, *p* ≤ 0.001) between 2002 and 2014 is consistent with the findings of other studies confirming the substantial increase in childhood obesity over the last decade [1,2,3,8,56]. In line with such findings [3,7,14,56], a higher prevalence of overweight and obesity was reported for boys *vs.* girls, and this increase was seen in each of the individual school grade categories. The literature is divided on this finding [4,8,10,11] and in some countries and contexts girls tended to be more overweight/obese than boys. Others have observed varying courses of development in overweight/obesity with peak values differing by age for boys and girls [4,7,9,10,14], as well as between different age categories of adolescents [4,8]. In line with the English obesity trends study [4] we found a higher prevalence of overweight/obesity among younger adolescents (5th grade) than in older adolescents (9th grade) (Table 2). This result underscores a disturbing finding that in developing countries the peak prevalence of obesity has moved from older adults to younger age cohorts [1]. However, other studies have found conversely higher prevalence of overweight/obesity in older adolescents than younger adolescents [8] and older children than younger children [6]. We found that amongst girls in all age categories, peaks in the occurrence of overweight and obesity were found in 2006, while for boys these peaks were in 2010. The consistency or slight decline in the reported prevalence of excessive body weight following the survey years 2010 and 2014 is of concern and remains a major health problem, especially in boys and the youngest age category of adolescents [7].

In terms of the study’s second objective, trends in the prevalence of young people who met PA recommendations were highlighted (MVPA—at least 60 min per day, VPA—at least 30 min and four or more days of the week). We confirmed a significant (*p* < 0.001) reduction in the odds of the overall group of adolescent boys and girls meeting MVPA recommendations in 2014 when compared with 2002. This finding is consistent with other studies [7,20,21,22,25] that have noted such declines, although a stable or even an increasing trend has been observed elsewhere [13,21,26] in self-reported MVPA [13,21,26] as well as objectively monitored PA over the past ten years [23,57].

The declines in meeting the daily MVPA recommendations between 2002 and 2014 mask the temporal patterns observed in the proportions of boys and girls meeting the VPA recommendations, as evident in former studies [7,13,20,21,22,23,57]. To illustrate, completely different trends in meeting VPA recommendations were found when comparing adolescent boys and girls between 2006 and 2014. Amongst boys, the proportion of those who met VPA recommendations significantly decreased (45.8%_2006_ to 42.2%_2014_, −3.6%, *p* < 0.001), while in girls we observed a significant increase in the proportion of those who met the VPA recommendations (24.4%_2006_ to 33.6%_2014_, +9.2%, *p* < 0.001). This finding is consistent with trends observed elsewhere, for example the higher growth observed in Icelandic adolescent girls engaging in VPA compared to boys, which may be associated with an increase in the active participation of girls in sports clubs between 1992 and 2006 [24]. Sports club training during early adolescence may counteract the decrease in PA reported for older adolescents [24,57]. In addition, frequent implementation of VPA is more often associated with good physical fitness and non-excessive body weight among adolescents [17,58,59,60]. Consistent with this idea, active training and VPA in sports clubs were associated with higher levels of fitness among children and adolescents [61,62] and a lower likelihood of excess body weight [63,64] than in non-sports club participants.

As regards objective three, we assessed whether the rapid increase in ST among boys and girls on weekdays as well as at weekends observed in the previous 2002–2010 surveys persisted in 2014. This study confirms the increase in the proportion of Czech adolescents with excessive ST (>2 h per day) between 2002 and 2014. Consistent with previous studies [5,26,27], this increase in the proportion of excessive ST was recorded on weekdays (boys: 75.1%_2002_ to 88.8%_2014_, +13.7%; girls: 61.3%_2002_ to 76.6%_2014_, +15.3%), as well as at weekends (boys: 78.3%_2002_–91.9%_2014_, +13.6%; girls: 65.1%_2002_ to 83.7%_2014_, +18.6%; all *p* < 0.001). However, unlike Brazilian [27], German [26], Chinese [28] and Portuguese adolescents [7], the highest proportion of excessive ST on weekdays and weekends among Czech adolescent girls was revealed in 2010, while in Czech adolescent boys the proportion of excessive ST continually grew up to 2014. Nevertheless, the current high levels of daily hours of ST reported on weekends among Czech adolescent boys (8.5 h per day) and girls (6.4 h per day) are a cause for serious concern when one takes the concerning prevalence of overweight/obesity into account. The finding that the highest proportion of excessive ST is in the oldest age group of the surveyed adolescents supports the contention of previous studies [26,65] that periods of sedentary behaviour increase as adolescents grow older.

A strong trend was revealed in the structure of screen time among Czech adolescents between 2002 and 2014 regardless of gender and type of day (weekdays *versus* weekend). Like the American [13,25], Brazilian [27], German [26], and Portuguese adolescents [7], the Czech adolescents also reported that time spent watching TV had declined slightly over the past 12 years, but conversely time spent on a PC had significantly increased. Thus, one kind of sedentary behaviour in adolescence had been replaced by another kind, and this trend that had emerged during recent years appears to be continuing [5]. However, a precise assessment of the amount and proportion of TV and PC screen time is difficult due to the fact that the self-administered HBSC questionnaire did not include an item on the time spent with a mobile phone (smartphone), which has recently become a common, easy and popular way to spend leisure time in adolescence [66,67], or multiple simultaneous ST.

In accordance with objective four, correlates of adolescents’ excess body weight were investigated. Consistent with previous studies that use objective accelerometers to monitor MVPA and sedentary behaviour among young people [17,65,68,69,70], we discovered that at least 60 min of daily MVPA and non-excessive ST (≤2 h per weekend day) was significantly associated with normal body weight status. In addition to the aforementioned studies [65,68,69,70], both variables (meeting the daily MVPA recommendation and non-excessive weekend ST) simultaneously reduced the likelihood of overweight/obesity in adolescents. However low levels of ST may not necessarily predict higher levels of PA [70,71]. In accordance with studies [68,69,70,71] we mention that ST and PA may be separate entities, each of them being associated with obesity in a different way. The association between excessive TV watching and clustered metabolic risk is mediated by adiposity, whereas lack of PA is associated with individual metabolic-risk indicators independently of obesity [70]. Therefore, in agreement with the conclusions of the above mentioned studies [68,69,70,71] the results of our study highlight the need for current intervention strategies aimed at reducing excessive body weight—a multilevel approach involving energy balance-related behaviour (PA, sedentary and dietary behaviour) in school and family-friendly environments [72,73,74,75]. A long-term multilevel approach targeted at increasing PA and reducing sedentary behaviour prevents excessive weight gain, not only during the course of multilevel interventions [34,74], but also more than two years after its cessation [35,75].

From a public health perspective, it is desirable to capture and analyse the trends in overweight/obesity among young people, because adolescence is the period of life where PA and ST behaviour patterns tend to be shaped, adopted, and transmitted into adulthood [31,32,33,76,77]. The present study extends the current literature on the trends in overweight/obesity and its correlates (MVPA, VPA and ST).

## 5. Strengths and Limitations

The main strengths of this study include the use of national representative data sets covering 12 years (2002–2014) with the application of the same procedures and protocol [37] by the same group of researchers over the four cycles of survey administration. The large sample size permitted the conduct of trend analyses separately among girls and boys in three age groups. A second strength is the usage of identical outcome variables dichotomized according to the same “cut-off points” in each survey cycle. Conversely, in the case of ST, splitting one wide-content question on PC use (2002) into two narrower questions on PC use (2006–2014) hypothetically could be a potential source of inaccuracy in determining the daily time of ST.

The main limitation of the study is the fact that the data are obtained on the basis of self-report. Although previous studies have demonstrated acceptable reliability and validity of self-reported measures of PA and ST for the past seven days [41,42,43,44,48,49,50], such self-reported measures are more prone to misclassification than objective measures [78,79]. For example, although direct measurement of body weight and height is more accurate for determining overweight/obesity than self-reporting, self-reporting of body weight and height is considered to be a reasonably valid tool for BMI estimation in epidemiological studies [39] for determining overweight and obesity [80,81]. However, a solid body of literature documents that self-reported data generally lead to underestimation of BMI, especially among girls and among overweight and obese adolescents [82]. Therefore, we can reasonably expect that the prevalence of overweight/obesity is actually even higher than indicated by the present study. However, in the Czech Republic, there is a serious debate on the way of rating overweight and obesity in adolescents especially by questionnaire-based research. Moreover, determination of overweight/obesity using an age-differentiated BMI percentile chart [38] without more accurate knowledge of body composition or current biological age of adolescents might complicate data interpretation for borderline individuals. Our dichotomous classification of all output trend variables is a less refined approach than more continuous or ordinal classification systems, however, such a classification is probably less prone to error. Finally, it should be emphasized that the presented findings refer to cross-sectional trends that may not accurately reflect the findings of true longitudinal study approaches.

## 6. Conclusions

Our major study findings highlight a disturbingly high level of overweight and obesity that has plateaued during recent cycles of the Czech HBSC study, especially among boys and in the youngest age category of adolescents. This has occurred coincidentally with a decrease in meeting the MVPA recommendations reported by boys, and consistent levels of meeting the MVPA recommendations reported by girls. Moreover, for the overall group of boys we found a rather alarming increase in excessive ST on weekdays, as well as at weekends, during the years 2002 to 2014 and a decrease in meeting the VPA recommendations between 2006 and 2014. Conversely, for girls, in 2014 we observed the cessation of past increases in the proportion of those with excessive ST on weekdays and weekends, while the number of girls meeting the VPA recommendations grew compared to 2006. The consistently exposed correlates of overweight and obesity of Czech adolescents between 2002 and 2014 are: less than 60 min of MVPA per day, more than 2 h per day of ST on weekdays, and younger age of adolescents.

Findings from this study could help to strengthen the current and upcoming efforts of government and commercial organizations in evaluating the effect of current intervention programmes aimed at reducing excessive body weight among Czech adolescents, especially in the youngest age groups [34,35]. For public health purposes, it is important to monitor the further development of the alarmingly high “plateau” in the prevalence of overweight and obesity among adolescents and whether this trend remains consistent with all adolescent age categories. Thus, it might be inspiring for researchers to clarify in greater depth the different trends in the development of VPA between boys and girls, and explore the possible effects of VPA on the reduction of excess body weight among adolescents.

## References

[1] Ng M., Fleming T., Robinson M., Thomson B., Graetz N., Margono C., Mullany E.C., Biryukov S., Abbafati C., Abera S.F. (2014). Global, regional, and national prevalence of overweight and obesity in children and adults during 1980–2013: A systematic analysis for the Global Burden of Disease Study 2013. Lancet.

[2] Dos Santos F.K., Maia J.A.R., Gomes T.N.Q.F., Daca T., Madeira A., Katzmarzyk P.T., Prista A. (2014). Secular trends in growth and nutritional status of Mozambican school-aged children and adolescents. PLos ONE.

[3] Van Nassau F., Singh A.S., Van Mechelen W., Brug J., Chinapaw M.J.M. (2014). Body mass index, waist circumference and skin-fold thickness in 12- to 14-year-old Dutch adolescents: Differences between 2006 and 2011. Pediatr. Obes..

[4] Stamatakis E., Zaninotto P., Falaschetti E., Mindel J., Head J. (2010). Time trends in childhood and adolescent obesity in England from 1995 to 2007 and projections of prevalence to 2015. J. Epidemiol. Community Health.

[5] Sigmundová D., Sigmund E., Hamrik Z., Kalman M. (2014). Trends of overweight and obesity, physical activity and sedentary behaviour in Czech schoolchildren: HBSC study. Eur. J. Public Health.

[6] Wijnhoven T.M.A., Van Raaij J.M.A., Spinelli A., Starc G., Hassapidou M., Spiroski I., Rutter H., Martos E., Rito A.I., Hovengen R. (2014). WHO European Childhood Obesity Surveillance Initiative: Body mass index and level of overweight among 6–9-year-old children from school year 2007/2008 to school year 2009/2010. BMC Public Health.

[7] Marques D., De Matos M.G. (2014). Trends and correlates of overweight and obesity among adolescents from 2002 to 2010: A three-cohort study on a representative sample of Portuguese adolescents. Am. J. Hum. Biol..

[8] Ogden C.L., Carroll M.D., Kit B.K., Flegal K.M. (2012). Prevalence of obesity and trends in body mass index among US children and adolescents, 1999–2010. J. Am. Med. Assoc..

[9] Olds T., Maher C., Zumin S., Péneau S., Lioret S., Castetbon K., Bellisle, de Wilde J., Hohepa M., Maddison R. (2011). Evidence that the prevalence of childhood overweight is plateauing: Data from nine countries. Int. J. Pediatr. Obes..

[10] Moraeus L., Lissner L., Sjöberg A. (2014). Stable prevalence of obesity in Swedish schoolchildren from 2008 to 2013 but widening socio-economic gap in girls. Acta Paediatr..

[11] Keane E., Kearney P.M., Perry I., Kelleher C.C., Harrington M. (2014). Trends and prevalence of overweight and obesity in primary school aged children in the Republic of Ireland from 2002–2012: A systematic review. BMC Public Health.

[12] Lissner L., Sohlström A., Sundblom E., Sjöberg A. (2010). Trends in overweight and obesity in Swedish schoolchildren 1999–2005: Has the epidemic reached a plateau?. Obes. Rev..

[13] Iannoti R.J., Wang J. (2013). Trends in physical activity, sedentary behaviour, diet, and BMI among US adolescents, 2001–2009. Pediatrics.

[14] Ahluwalia N., Dalmasso P., Rasmussen M., Lipsky L., Currie C., Haug E., Kelly C., Damsgaard M.T., Due P., Tabak I. (2014). Trends in overweight prevalence among 11-, 13- and 15-year-olds in 25 countries in Europe, Canada and USA from 2002 to 2010. Eur. J. Public Health.

[15] Katzmarzyk P.T., Barreira T.V., Broyles S.T., Champagne C.M., Chaput J.P., Fogelholm M., Hu G., Johnson W.D., Kuriyan R., Kurpad A. (2013). The international study of childhood obesity, lifestyle and the environment (ISCOLE): Design and methods. BMC Public Health.

[16] Parikh T., Stratton G. (2011). Influence of intensity of physical activity on adiposity and cardiorespiratory fitness in 5–18 year olds. Sports Med..

[17] Ness A.R., Leary S.D., Mattocks C., Blair S.N., Reilly J.J., Wells J., Ingle S., Tilling K., Smith G.D., Riddoch C. (2007). Objectively measured physical activity and fat mass in a large cohort of children. PLos Med..

[18] White J., Jago R. (2012). Prospective associations between physical activity and obesity among adolescent girls. Arch. Pediatr. Adolesc. Med..

[19] Lissner L., Lanfer A., Gwozdz W., Olafsdottir S., Eiben G., Moreno L.A., Santaliestra-Pasías A.M., Kovács E., Barba G., Loit H.M. (2012). Television habits in relation to overweight, diet and taste preferences in European children: The IDEFICS study. Eur. J. Epidemiol..

[20] Sigmundová D., El Ansari W., Sigmund E., Frömel K. (2011). Secular trends: A ten-year comparison of the amount and type of physical activity and inactivity of random samples of adolescents in the Czech Republic. BMC Public Health.

[21] Kalman M., Inchley J., Sigmundová D., Iannotti R.J., Tynjälä J.A., Hamrik Z., Haug E., Bucksch J. (2015). Secular trends in moderate-to-vigorous physical activity in 32 countries from 2002 to 2010: A cross-national perspective. Eur. J. Public Health.

[22] Dos Santos F.K., Maia J.A.R., Gomes T.N.Q.F., Daca T., Madeira A., Damasceno A., Katzmarzyk P.T., Prista A. (2014). Secular trends in habitual physical activities of Mozambican children and adolescents from Maputo city. Int. J. Environ. Res. Public Health.

[23] Raustorp A., Ekroth Y. (2010). Eight-year secular trends of pedometer-determined physical activity in young Swedish adolescents. J. Phys. Act. Health.

[24] Eithdóttir S.T., Kristjánsson Á.L., Sigfúsdóttir I.D., Allegrante J.P. (2008). Trends in physical activity and participation in sports clubs among Icelandic adolescents. Eur. J. Public. Health.

[25] Bassett D.R., John D., Conger S.A., Fitzhugh E.C., Coe D.P. (2014). Trends in physical activity and sedentary behaviors of U.S. youth. J. Phys. Act. Health.

[26] Bucksch J., Inchley J., Hamrik Z., Finne E., Kolip Z., HBSC Study Group Germany (2014). Trends in television time, non-gaming PC use and moderate-to-vigorous physical activity among German adolescents 2002–2010. BMC Public Health.

[27] Lopes A.S., Silva K.S., Filho V.C.B., Bezerra J., De Oliveira E.S.A., Nahas M.V. (2014). Trends in screen time on week and weekend days in a representative sample of Southern Brazil students. J. Public Health.

[28] Cui Z., Hardy L.L., Dibley M.J., Bauman A. (2011). Temporal trends and recent correlates in sedentary behaviours in Chinese children. Int. J. Behav. Nutr. Phys. Act..

[29] Lehnert T., Sonntag D., Konnopka A., Riedel-Heller S., König H.H. (2013). Economic costs of overweight and obesity. Best Pract. Res. Clin. Endocrinol. Metable.

[30] Von Lengerke T., Krauth C. (2011). Economic costs of adult obesity: A review of recent European studies with a focus on subgroup-specific costs. Maturitas.

[31] Herman K.M., Craig C.L., Gauvin L., Katzmarzyk P.T. (2009). Tracking of obesity and physical activity from childhood to adulthood: The Physical Activity Longitudinal Study. Int. J. Pediatr. Obes..

[32] Wennberg P., Gustafsson P.E., Howard B., Wennberg M., Hammarström A. (2014). Television viewing over the life course and the metabolic syndrome in mid-adulthood: A longitudinal population-based study. J. Epidemiol. Community Health.

[33] Rangul V., Bauman A., Holmen T.L., Midthjell K. (2012). Is physical activity maintenance from adolescence to young adulthood associated with reduced CVD risk factors, improved mental health and satisfaction with life: The HUNT Study, Norway. Int. J. Behav. Nutr. Phys. Act..

[34] Sigmund E., El Ansari W., Sigmundová D. (2012). Does school-based physical activity decrease overweight and obesity in children aged 6–9 years? A two-year non-randomized longitudinal intervention study in the Czech Republic. BMC Public Health.

[35] Sigmund E., Sigmundová D. (2013). Longitudinal 2-year follow-up on the effect of a non-randomised school-based physical activity intervention on reducing overweight and obesity of Czech children aged 10–12 years. Int. J. Environ. Res. Public Health.

[36] Webber L., Kilpi F., Marsh T., Rtveladze K., McPherson K., Brown M. (2012). Modelling obesity trends and related diseases in Eastern Europe. Obes. Rev..

[37] Currie C., Nic Gabhainn S., Godeau E., The International HBSC Network Coordinating Committee (2009). The Health Behaviour in School-aged Children. WHO Collaborative Cross-National (HBSC) study: Origins, concept, history and development 1982–2008. Int. J. Public Health.

[38] Roberts C., Freeman J., Samdal O., Schnohr C.W., Looze M., Nic Gabhainn S., Iannotti R., Rasmussen M., the International HBSC Study Group (2009). The Health Behaviour in School-aged Children (HBSC) study: Methodological developments and current tensions. Int. J. Public Health.

[39] Fonseca H., Silva A.M., Matos M.G., Esteves I., Costa P., Guerra A., Gomes-Pedro J. (2010). Validity of BMI based on self-reported weight and height in adolescents. Acta Paediatr..

[40] WHO Growth Reference Data for 5–19 Years. WHO Reference 2007. http://www.who.int/growthref/en.

[41] Prochaska J.J., Sallis J.F., Long B. (2001). A physical activity screening measure for use with adolescents in primary care. Arch. Pediatr. Adolesc. Med..

[42] Ridgers N.D., Timperio A., Crawford D., Salmon J. (2012). Validity of a brief self-report instrument for assessing compliance with physical activity guidelines amongst adolescents. J. Sci. Med. Sport.

[43] Liu Y., Wang M., Tynjälä J., Lv Y., Villberg J., Zhang Z., Kannas L. (2010). Test-retest reliability of selected items of Health Behaviour in School-aged Children (HBSC) survey questionnaire in Beijing, China. BMC Med. Res. Methodol..

[44] Bobakova D., Hamrik Z., Badura P., Sigmundova D., Nalecz H., Kalman M. (2015). Test-retest reliability of selected physical activity and sedentary behaviour HBSC items in the Czech Republic, Slovakia and Poland. Int. J. Public Health.

[45] Janssen I., Leblanc A.G. (2011). Systematic review of the health benefits of physical activity and fitness in school-aged children and youth. Int. J. Behav. Nutr. Phys. Act..

[46] Tremblay M.S., Warburton D.E.R., Janssen I., Paterson D.H., Latimer A.E., Rhodes R.E., Kho M.E., Hicks A., LeBlanc A.G., Zehr L. (2011). New Canadian physical activity guidelines. Appl. Physiol. Nutr. Metable.

[47] World Health Organization Global Recommendations on Physical Activity for Health. Geneva, Switzerland: World Health Organization, 2010. http://www.who.int/dietphysicalactivity/factsheet_recommendations/en/.

[48] Schmitz K.H., Harnack L., Fulton J.E., Jacobs D.R., Gao S., Lytle L.A., Van Coevering P. (2004). Reliability and validity of a brief questionnaire to assess television viewing and computer use by middle school children. J. Sch. Health.

[49] Vereecken C.A., Todd J., Roberts C., Mulvihill C., Maes L. (2006). Television viewing behaviour and associations with food habits in different countries. Public Health Nutr..

[50] Rey-López J.P., Vicente-Rodriguez G., Ortega F.B., Ruiz J.R., Martinez-Gómez D., De Henauw S., Manios Y., Molnar D., Polito A., Verloigne M. (2010). Sedentary patterns and media availability in European adolescents: The HELENA study. Prev. Med..

[51] Lau D.C.W., Douketis J.D., Morrison K.M., Hramiak I.M., Sharma A.M., Ur E. (2007). Obesity Canada Clinical Practice Expert Panel. 2006 Canadian clinical practice guidelines on the management and prevention of obesity in adults and children [summary]. Can. Med. Assoc. J..

[52] Committee on Public Education (2001). Children, adolescents, and television. Pediatrics.

[53] Tremblay M.S., LeBlanc A.G., Kho M.E., Saunders T.J., Larouche R., Colley R.C., Goldfield G., Gorber S.C. (2011). Systematic review of sedentary behaviour and health indicators in school-aged children and youth. Int. J. Behav. Nutr. Phys. Act..

[54] Branca F., Nikogosian H., Lobstein T. (2007). The Challenge of Obesity in the WHO European Region and the Strategies for Response: Summary. http://www.euro.who.int/__data/assets/pdf_file/0008/98243/E89858.pdf?ua=1.

[55] Knai C., Suhrcke M., Lobstein T. (2007). Obesity in Eastern Europe: An overview of its health and economic implication. Econ. Hum. Biol..

[56] Liang Y.J., Xi B., Song A.Q., Liu J.X., Mi J. (2012). Trends in general and abdominal obesity among Chinese children and adolescents 1993–2009. Pediatr. Obes..

[57] Raustorp A., Svensson K., Perlinger T. (2007). Tracking of pedometer-determined physical activity: A 5-year follow-up study of adolescents in Sweden. Pediatr. Exerc. Sci..

[58] Lätt E., Mäestu J., Ortega F.B., Rääsk T., Jürimäe T., Jürimäe J. (2015). Vigorous physical activity rather than sedentary behaviour predicts overweight and obesity in pubertal boys: A 2-year follow-up study. Scan. J. Public Health.

[59] Guttin B., Yin Z., Humphries M.C., Barbeau P. (2005). Relations of moderate and vigorous physical activity to fitness and fatness in adolescents. Am. J. Clin. Nutr..

[60] Patrick K., Norman G.J., Calfas K.J., Sallis J.F., Zabinski M.F., Rupp J., Cella J. (2004). Diet, physical activity, and sedentary behaviors as risk factors for overweight in adolescence. Arch. Pediatr. Adolesc. Med..

[61] Zahner L., Muehlbauer T., Schmid M., Meyer U., Puder J.J., Kriemler S. (2009). Association of sports club participation with fitness and fatness in children. Med. Sci. Sports Exerc..

[62] Golle K., Granacher U., Hoffman M., Wick D., Muehlbauer T. (2014). Effect of living area and sports club participation on physical fitness in children: A 4 year longitudinal study. BMC Public Health.

[63] Drenowatz C., Steiner R.P., Brandstetter S., Klenk J., Wabitsch M., Steinacker M. (2013). Organized sport, overweight, and physical fitness in primary school children in Germany. J. Obes..

[64] Basterfield L., Reilly J.K., Pearce M.S., Parkinson K.N., Adamson A.J., Reilly J.J., Vella S.A. (2014). Longitudinal associations between sports participation, body composition and physical activity from childhood to adolescence. J. Sci. Med. Sport.

[65] Mitchell J.A., Pate R.R., Nader P.R. (2013). Time spent in sedentary behavior and changes in childhood BMI: A longitudinal study from ages 9 to 15 years. Int. J. Obes..

[66] Madden M., Lenhart A., Duggan M., Cortesi S., Gasser U. Teen and Technology. Washington, DC: Pew Internet & American Life Project, 2013. http://www.pewinternet.org/Reports/2013/Teens-and-Tech.aspx..

[67] Hysing M., Pallesen S., Stormark K.M., Jakobsen R., Lundervold A.J., Sivertsen B. (2015). Sleep and use of electronic devices in adolescence: Results from a large population-based study. BMJ Open.

[68] Ekelund U., Luan J., Sherar L.B., Esliger D.W., Griew P., Cooper A. (2012). Moderate to vigorous physical activity and sedentary time and cardiometabolic risk factors in children and adolescents. J. Am. Med. Assoc..

[69] Chaput J.P., Saunders T.J., Mathieu M.-È., Henderson M., Tremblay M.S., O’Loughlin J., Tremblay A. (2013). Combined associations between moderate to vigorous physical activity and sedentary behaviour with cardiometabolic risk factors in children. Appl. Physiol. Nutr. Metab.

[70] Ekelund U., Brage S., Froberg K., Harro M., Anderssen S.A., Sardinha L.B., Riddoch C., Andersen L.B. (2006). TV viewing and physical activity are independently associated with metabolic risk in children: The European Youth Heart Study. PLoS. Med..

[71] Fakhouri T.H.I., Hughes J.P., Brody J.D., Kit B.K., Ogden C.L. (2013). Physical activity and screen-time viewing among elementary school-aged children in the United States from 2009 to 2010. JAMA Pediatr..

[72] Brug J., Te Velde S.J., Chinapaw M.J.M., Bere E., De Bourdeaudhuij I.M.M., Moore H., Maes L., Jensen J., Manios Y., Lien N. (2010). Evidence-based development of school-based and family-involved prevention of overweight across Europe: The ENERGY-project’s design and conceptual framework. BMC Public Health.

[73] Brug J., Van Stralen M.M., Te Velde S.J., Chinapaw M.J.M., De Bourdeaudhuij I., Lien N., Bere E., Maskini V., Singh A.S., Maes L. (2014). Difference in weight status and energy-balance related behaviors among schoolchildren across Europe: The ENERGY-project. PLoS ONE.

[74] Simon C., Schweitzer B., Oujaa M., Wagner A., Arveiler D., Triby E., Copi N., Blanc S., Platat C. (2008). Successful overweight prevention in adolescents by increasing physical activity: A 4-year randomized controlled intervention. Int. J. Obes..

[75] Simon C., Kellou N., Dugas J., Platat C., Copin N., Schweitzer B., Hausser F., Bergouignan A., Lefai E., Blanc S. (2014). A socio-ecological approach promoting physical activity and limiting sedentary behaviour in adolescence showed weight benefits maintained 2.5 years after intervention cessation. Int. J. Obes..

[76] Telama R., Yang X., Viikari J., Välimäki I., Wanne O., Raitakari O. (2005). Physical activity from childhood to adulthood: A 21-year tracking study. Am. J. Prev. Med..

[77] Craigie A.M., Lake A.A., Kelly S.A., Adamson A.J., Mathers J.C. (2011). Tracking of obesity-related behaviours from childhood to adulthood: A systematic review. Maturitas.

[78] Adamo K.B., Prince S.A., Tricco A.C., Connor-Gorber S., Tremblay M. (2009). A comparison of indirect *versus* direct measures for assessing physical activity in the pediatric population: A systematic review. Int. J. Pediatr. Obes..

[79] Armstrong N., Welsman J.R. (2006). The physical activity patterns of European youth with reference to methods of assessment. Sports Med..

[80] Lindsay R.S., Hanson R.L., Roumain J., Ravussin E., Knowler W.C., Tataranni P.A. (2001). Body mass index as a measure of adiposity in children and adolescents: Relationship to adiposity by dual energy X-ray absorptiometry and to cardiovascular risk factors. J. Clin. Endocrinol. Metab..

[81] Freedman D.S., Sherry B. (2009). The validity of BMI as an indicator of body fatness and risk among children. Pediatrics.

[82] Elgar F.J., Stewart J.M. (2008). Validity of self-report screening for overweight and obesity: Evidence from the Canadian Community Health Survey. Can. J. Public. Health.

